# Bis[2-(4,5-diphenyl-1*H*-imidazol-2-yl)-4-nitrophenolato]copper(II) dihydrate: crystal structure and Hirshfeld surface analysis

**DOI:** 10.1107/S2056989019013720

**Published:** 2019-10-22

**Authors:** Sailesh Chettri, Dhiraj Brahman, Biswajit Sinha, Mukesh M. Jotani, Edward R. T. Tiekink

**Affiliations:** a Department of Chemistry, St. Joseph’s College, Darjeeling 734 104, India; b Department of Chemistry, University of North Bengal, Darjeeling 734 013, India; c Department of Physics, Bhavan’s Sheth R. A. College of Science, Ahmedabad, Gujarat 380 001, India; d Research Centre for Crystalline Materials, School of Science and Technology, Sunway University, 47500 Bandar Sunway, Selangor Darul Ehsan, Malaysia

**Keywords:** crystal structure, copper(II), coordination complex, Hirshfeld surface analysis, computational chemistry

## Abstract

The crystal and mol­ecular structures of the title Cu^II^ complex, isolated as a dihydrate, [Cu(C_21_H_14_N_3_O_3_)_2_]·2H_2_O, reveals a highly distorted coordination geometry inter­mediate between square-planar and tetra­hedral defined by an N_2_O_2_ donor set derived from two mono-anionic bidentate ligands. Furthermore, each six-membered chelate ring adopts an envelope conformation with the Cu atom being the flap. In the crystal, imidazolyl-amine-N—H⋯O(water), water-O—H⋯O(coordinated, nitro and water), phenyl-C—H⋯O(nitro) and π(imidazol­yl)–π(nitro­benzene) [inter-centroid distances = 3.7452 (14) and 3.6647 (13) Å] contacts link the components into a supra­molecular layer lying parallel to (101). The connections between layers forming a three-dimensional architecture are of the types nitro­benzene-C—H⋯O(nitro) and phenyl-C—H⋯π(phen­yl). The distorted coordination geometry for the Cu^II^ atom is highlighted in an analysis of the Hirshfeld surface calculated for the metal centre alone. The significance of the inter­molecular contacts is also revealed in a study of the calculated Hirshfeld surfaces; the dominant contacts in the crystal are H⋯H (41.0%), O⋯H/H⋯O (27.1%) and C⋯H/H⋯C (19.6%).

## Abstract 2

A coordination geometry inter­mediate between square-planar and tetra­hedral, defined by an N_2_O_2_ donor set, is found in the title Cu^II^ complex. Conventional O—H⋯O and N—H⋯O hydrogen bonding leads to a supra­molecular layer in the crystal.

## Chemical context   

The title copper(II) complex, (I), was isolated during an on-going research programme on the catalytic activity of copper borate (CuB_4_O_7_) for C—N heterocyclic bond formation reactions. Complex (I) was formed during the attempted synthesis of a tri­aryl­imidazole derivative using benzil and the respective aromatic aldehyde with copper borate, using ammonium acetate as a nitro­gen source. The single-crystal analysis of the synthesized product revealed that in the copper(II) complex, the tri­aryl­imidazole moiety acts as a bidentate ligand for the copper atom. During the successful synthesis of the tri­aryl­imidazole, the desired product formed in good yield at a temperature in the range 100–110 °C. However, when the reaction was conducted at 130 °C and above, the title copper(II) complex formed instead of the targeted tri­aryl­imidazole. The crystal and mol­ecular structures of (I) are described herein, along with a detailed analysis of the mol­ecular packing *via* an analysis of the calculated Hirshfeld surfaces.

## Structural commentary   

**Figure 1:**
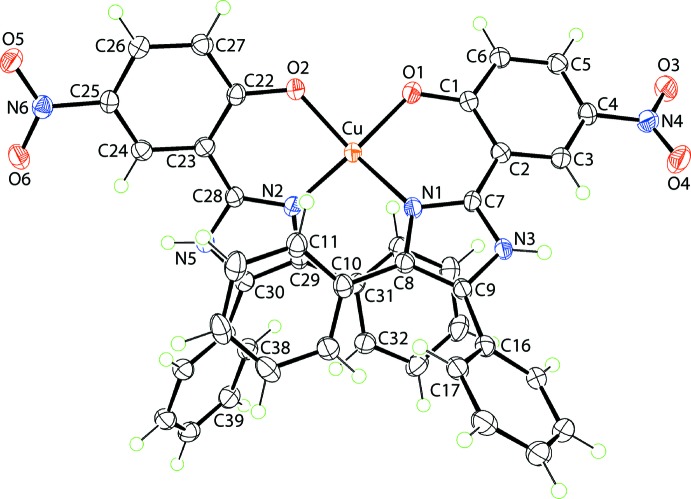
The mol­ecular structure of the complex mol­ecule in (I), showing the atom-labelling scheme and with displacement ellipsoids drawn at the 70% probability level.

**Figure 2:**
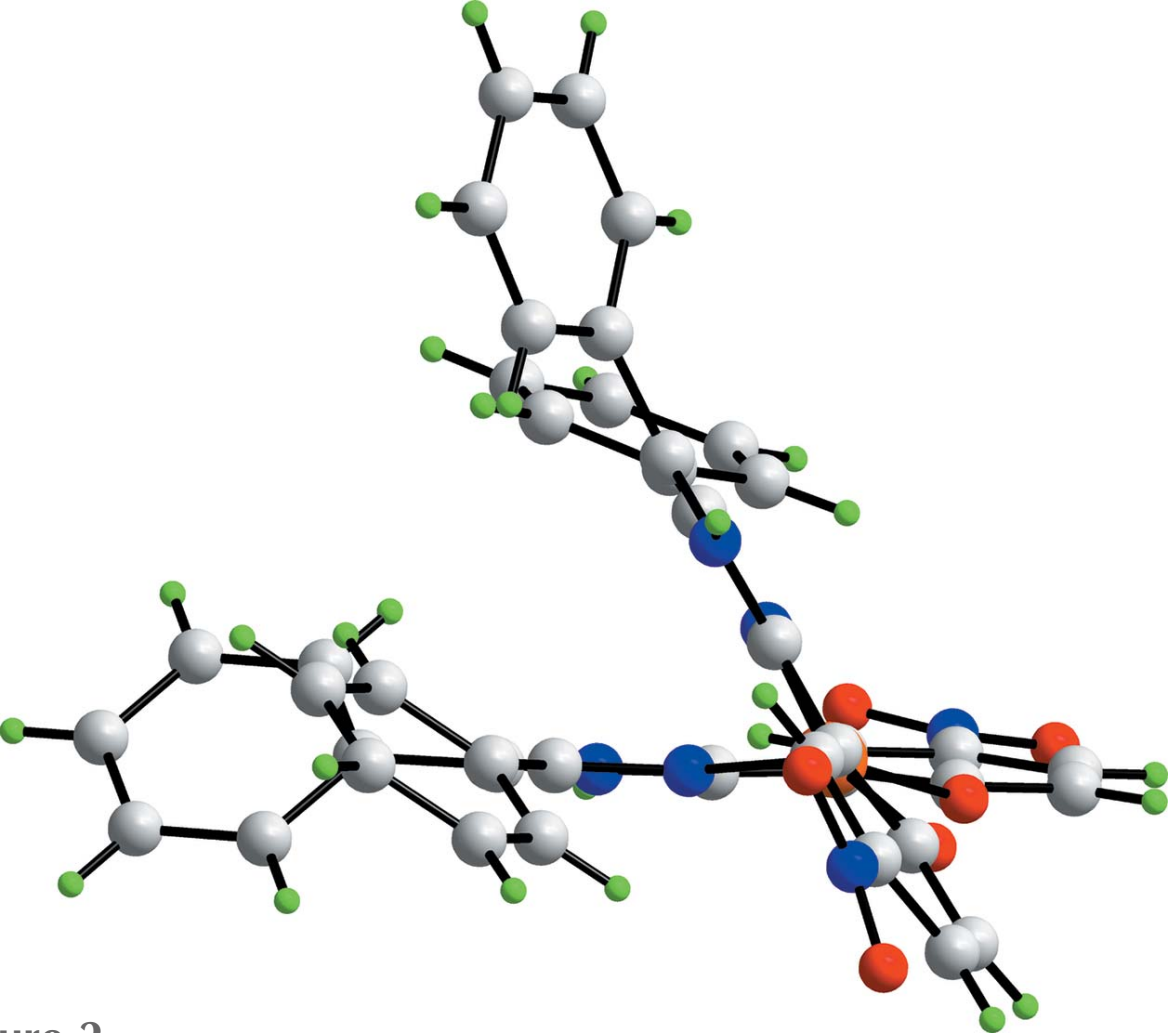
A view of the mol­ecular structure of the complex mol­ecule in (I), highlighting the distorted coordination geometry about the copper(II) atom.

The crystallographic asymmetric unit of (I) comprises a complex mol­ecule and two water mol­ecules of crystallization. The copper(II) centre in (I), Fig. 1 ▸, is bis-*N*,*O*-chelated by two 2-(4,5-diphenyl-1*H*-imidazol-2-yl)-4-nitrophenolate mono-anions. The resulting N_2_O_2_ donor set defines a highly distorted coordination geometry, as seen in the angles included in Table 1 ▸ and in the view of Fig. 2 ▸. The angles range from a narrow 89.36 (7)°, for O1—Cu—O2, to a wide 147.34 (8)°, for O1—Cu—N2. The distortion is highlighted in the dihedral angle between the best planes through the two chelate rings of 49.82 (7)°. The value of τ_4_ is a geometric measure of the distortion of a four-coordinate geometry (Yang *et al.*, 2007 ▸). For (I), the value computes as 0.48 which is almost exactly inter­mediate between the values of τ_4_ = 0 for an ideal tetra­hedron and τ_4_ = 1.0 for an ideal square-planar geometry. In fact, the six-membered chelate rings are not planar, each adopting an envelope conformation with the Cu atom being the flap atom. In this description, the r.m.s. deviation for the least-squares plane through the O1/N1/C1/C2 atoms is 0.036 Å with the Cu atom lying 0.410 (3) Å out of the plane. The comparable parameters for the O2-chelate ring are 0.033 and 0.354 (3) Å, respectively. The dihedral angle formed between the two planar regions of the chelate rings is 49.38 (8)°. The dihedral angles between the best plane through the O1-chelate ring and each of the fused six- and five-membered rings are 9.18 (12) and 5.54 (14)°, respectively; the equivalent angles for the O2-chelate rings are 8.44 (8) and 2.71 (9)°, respectively. The N1-imidazol-2-yl ring forms dihedral angles of 41.20 (11) and 37.46 (10)° with the C10- and C16-phenyl substituents, respectively, and the dihedral angle between the phenyl rings is 59.92 (8)°, *i.e*. all indicating splayed relationships. A similar situation pertains to the N2-imidazol-2-yl ring, where the comparable dihedral angles formed with the C31- and C37-phenyl rings are 38.29 (10), 48.5 (9) and 50.84 (7)°, respectively. Finally, the nitro groups are not strictly coplanar with the benzene rings to which they are connected, as seen in the dihedral angles of 14.2 (4)° for C1–C6/N4/O3/O4 and 5.9 (3)° for C22–C27/N6/O5/O6.
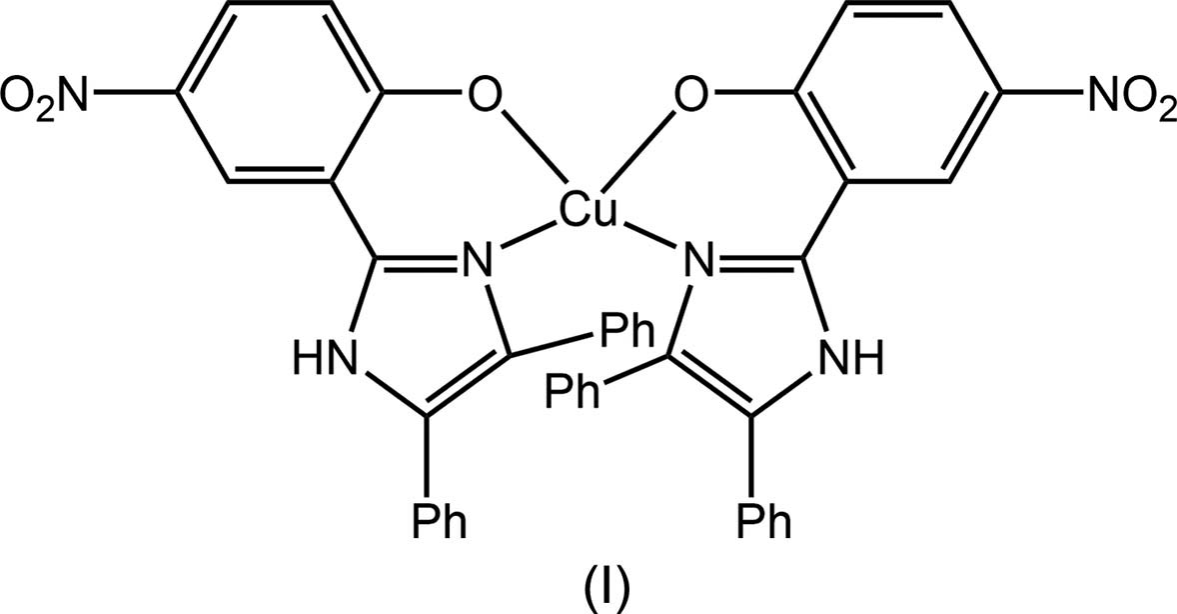


## Supra­molecular features   

**Figure 3:**
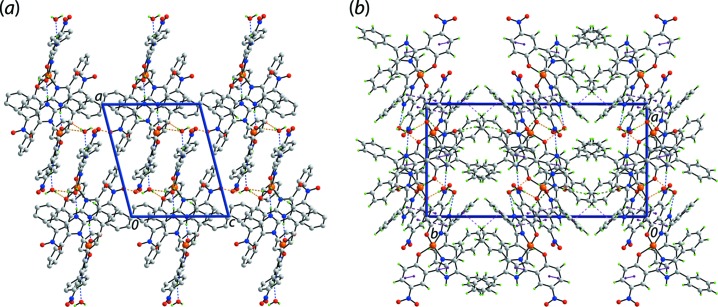
The mol­ecular packing in the crystal of (I): (*a*) a supra­molecular layer parallel to (101) sustained by O—H⋯O, N—H⋯O and C—H⋯O inter­actions shown as orange, blue and green dashed lines, respectively, and (*b*) a view of the unit-cell contents in projection down the *c* axis, with π–π and C—H⋯π inter­actions shown as purple and pink dashed lines, respectively.

As each component of the asymmetric unit has hydrogen-bonding functionality, conventional hydrogen bonds are found in the crystal of (I); the geometric parameters characterizing the identified inter­molecular inter­actions operating in the crystal of (I) are collated in Table 2 ▸. Each of the imidazolyl-amine-N—H atoms forms a donor inter­action to a water mol­ecule to generate a three-mol­ecule aggregate. The O1*W* water mol­ecule forms donor inter­actions to the coordinated O2 atom and to a symmetry-related O2*W* water mol­ecule. The O2*W* water mol­ecule connects to the coordinated O1 atom as well as to a nitro-O3 atom. Hence, the O2*W* water mol­ecule is involved in four hydrogen-bonding inter­actions. The fourth contact involving the O1*W* water mol­ecule, a C—H⋯O acceptor contact, is provided by the nitro­benzene ring. There is also a phenyl-C—H⋯O(nitro) contact of note, Table 2 ▸. The aforementioned inter­actions combine to stabilize a supra­molecular layer lying parallel to (101), as shown in Fig. 3 ▸(*a*). There are also π–π stacking and C—H⋯O inter­actions in the crystal, Fig. 3 ▸(*b*). Within layers, there are π–π inter­actions occurring between the imidazolyl and nitro­benzene rings [inter-centroid distances: *Cg*(N1/N3/C7–C9)⋯*Cg*(C1–C6) = 3.7452 (14) Å and angle of inclination = 9.70 (13)° for symmetry operation (−*x* + 2, −*y* + 1, −*z* + 1); *Cg*(N2/N5/C28–C30)⋯*Cg*(C22–C27) = 3.6647 (13) Å and angle of inclination = 8.15 (12)° for (−*x* + 1, −*y* + 1, −*z* + 1)]. The connections between layers along [010] are of the type nitro­benzene-C—H⋯O(nitro) and phenyl-C—H⋯π(phen­yl), as detailed in Table 2 ▸.

## Hirshfeld surface analysis   

**Figure 4:**
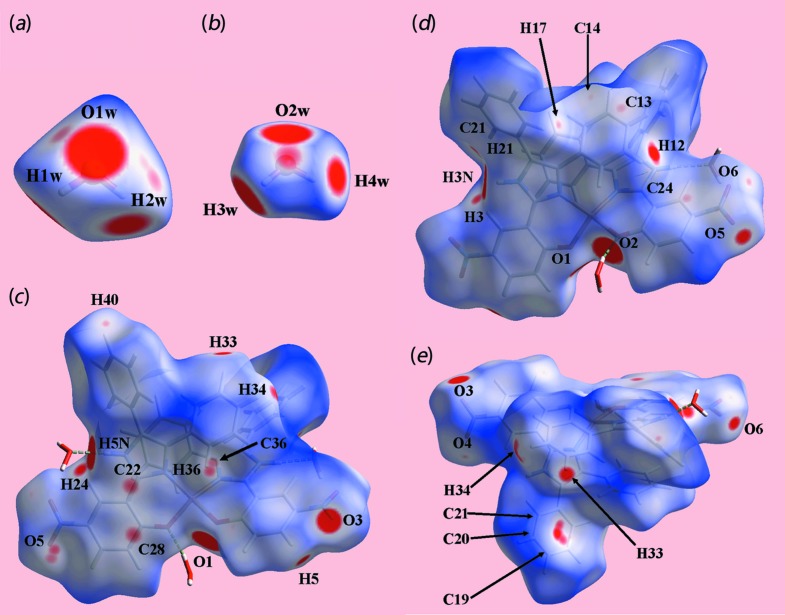
Different views of the Hirshfeld surfaces for the constituents of (I) mapped over *d*_norm_ for the (*a*) water-O1*W* mol­ecule [in the range −0.2369 to +1.2173 arbitrary units (au)], (*b*) water-O2*W* mol­ecule (−0.2114 to + 0.7500 au) and (*c*)–(*e*) complex mol­ecule (−0.1170 to + 1.6287 au).

The Hirshfeld surface calculations for (I) were performed with *CrystalExplorer17* (Turner *et al.*, 2017 ▸) and published protocols (Tan *et al.*, 2019 ▸), and serve to indicate the significant role of the two water mol­ecules in the supra­molecular association in the crystal. The involvement of both the water mol­ecules in hydrogen bonds, Table 2 ▸, are evident as bright-red spots near the respective atoms on the Hirshfeld surfaces mapped over *d*_norm_ for the O1*W*-, Fig. 4 ▸(*a*), and O2*W*-water, Fig. 4 ▸(*b*), mol­ecules. In addition, the presence of faint-red spots near the O1*W*, O2*W* and H1*W* atoms in Figs. 4 ▸(*a*) and 4(*b*) are indicative of the other contacts of these atoms with those of the Cu^II^ complex mol­ecule (Table 2 ▸). The donors and acceptors of the hydrogen bonds involving atoms of the complex mol­ecule are also apparent as bright-red spots near the participating atoms in the views of the Hirshfeld surfaces calculated for the complex mol­ecule shown in Figs. 4 ▸(*c*)–(*e*).

**Figure 5:**
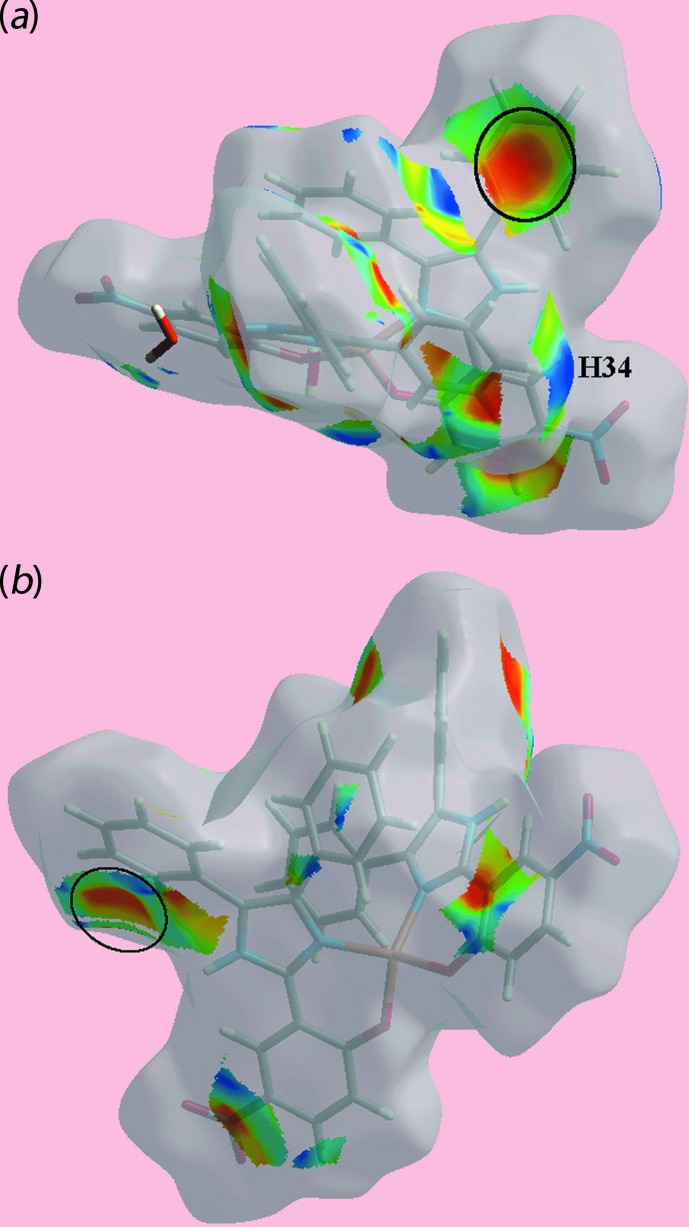
Two views of the Hirshfeld surface mapped with the shape-index property for the complex mol­ecule in (I) from −1.0 to +1.0 arbitrary units highlighting (*a*) the donor and acceptor atoms of the C—H⋯π inter­action through a blue bump near the H34 atom and bright-orange curvature, enclosed within the black circle, and (*b*) the O2*W*—H4*W*⋯π inter­action by the bright-orange region enclosed within the black circle.

**Figure 6:**
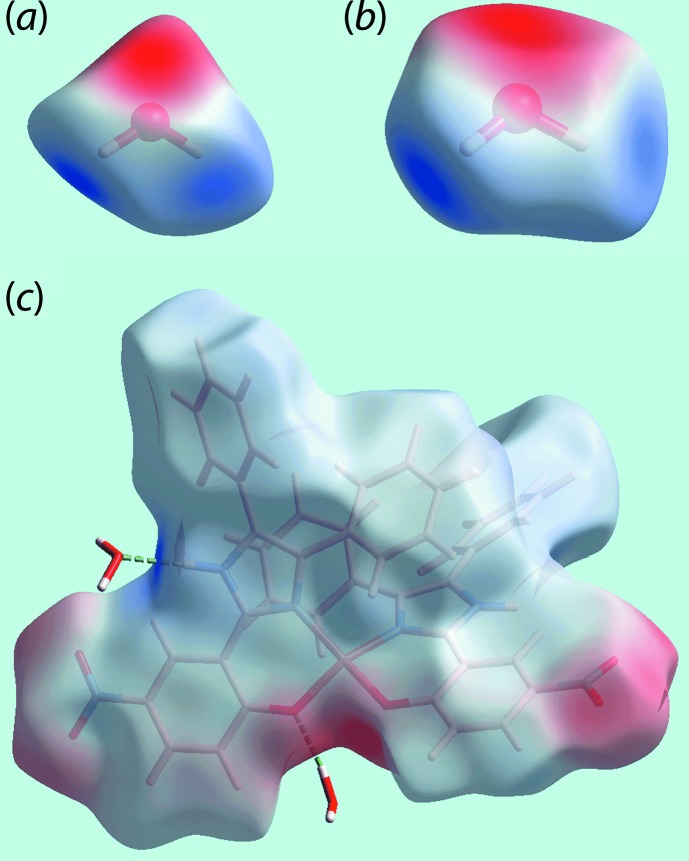
Different views of the Hirshfeld surfaces for the constituents of (I) mapped over the electrostatic potential (the red and blue regions represent negative and positive electrostatic potentials, respectively) for the (*a*) water-O1*W* mol­ecule [in the range −0.1001 to +0.1943 atomic units (a.u.)], (*b*) water-O2*W* mol­ecule (−0.1013 to +0.1751 a.u.) and (*c*) complex mol­ecule (−0.1209 to +0.2076 a.u.). The hydrogen bonds involving water mol­ecules in (*c*) are indicated by green dashed lines.

**Figure 7:**
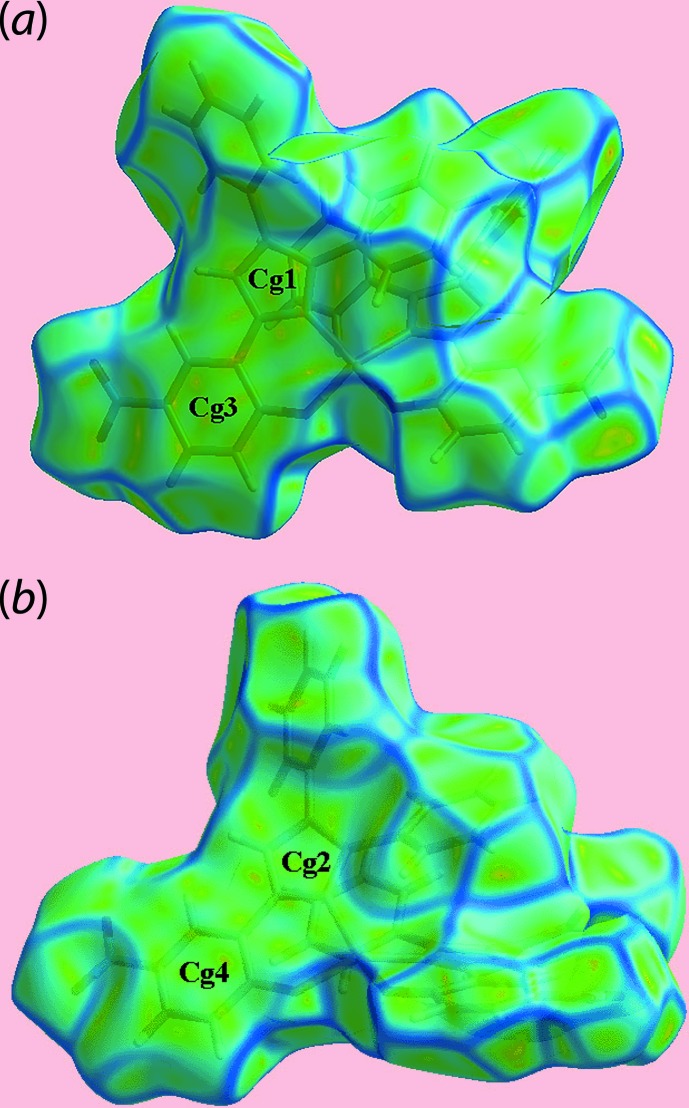
Two views of the Hirshfeld surface mapped over curvedness for the complex mol­ecule in (I), highlighting flat regions enclosing symmetry-related imidazole and nitro­benzene rings involved in π–π stacking, labelled *Cg*1 and *Cg*3 for one pair of rings in (*a*), and *Cg*2 and *Cg*4 for the other pair in (*b*).

The presence of a short inter­atomic C⋯C contact between atoms C22 and C28 (Table 3 ▸) arises from π–π stacking between symmetry-related imidazole and nitro­benzene rings, and is observable as the faint-red spots near these atoms on the *d*_norm_-mapped Hirshfeld surface in Fig. 4 ▸(*c*). The pair of faint-red spots appearing near the phenyl-C36 and H36 atoms, and also near the nitro-O5 atom on the surface indicating short inter­atomic contacts that characterize the weak C—H⋯O inter­action, Table 3 ▸. The influence of the C—H⋯π contact on the mol­ecular packing is recognized from the three faint-red spots in the phenyl-(C16–C21) ring and another near atom H34 in Fig. 4 ▸(*e*). The donors and acceptors of this inter­action are also evident as the blue bump and a bright-orange spot enclosed within the black circle on the Hirshfeld surface mapped with the shape-index property in Fig. 5 ▸(*a*). The bright-orange region enclosed within a black circle in Fig. 5 ▸(*b*) is also an indication of the O2*W*—H4*W*⋯*Cg*(C16–C21) contact. The Hirshfeld surfaces mapped over the calculated electrostatic potential for the water and complex mol­ecules in Fig. 6 ▸ also illustrate the donors and acceptors of inter­molecular inter­actions through blue and red regions corresponding to positive and negative electrostatic potentials, respectively. The π–π stacking between symmetry-related imidazolyl and nitro­benzene rings are viewed as the flat regions enclosing them on the Hirshfeld surfaces mapped over curvedness in Fig. 7 ▸. On the Hirshfeld surfaces mapped over *d*_norm_ illustrated in Figs. 4 ▸(*c*)–(*e*), faint-red spots also appear near other atoms indicating their involvement in other short inter­atomic contacts, as summarized in Table 3 ▸.

**Figure 8:**
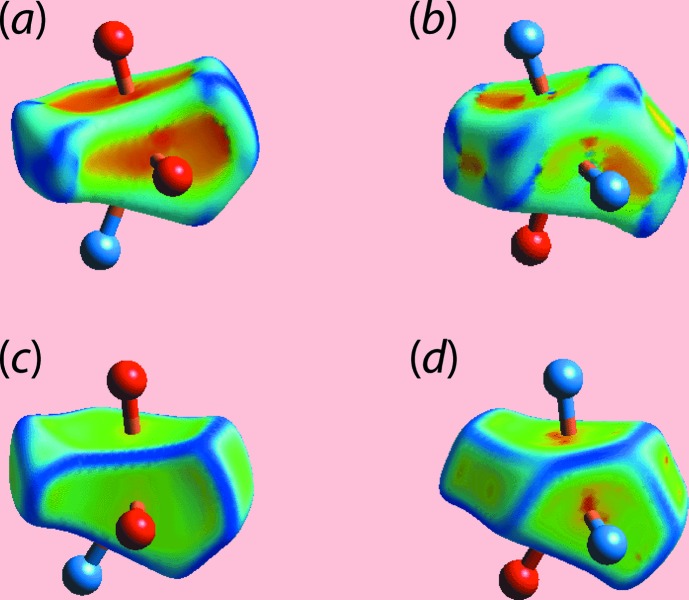
Different views of the Hirshfeld surfaces calculated for the copper(II) centre in (I) highlighting the coordination by the N_2_O_4_ donor set mapped over (*a*)/(*b*) shape-index in the range −1.0 to +1.0 arbitrary units and (*c*)/(*d*) curvedness in the range −4.0 to +0.4 arbitrary units.

**Figure 9:**
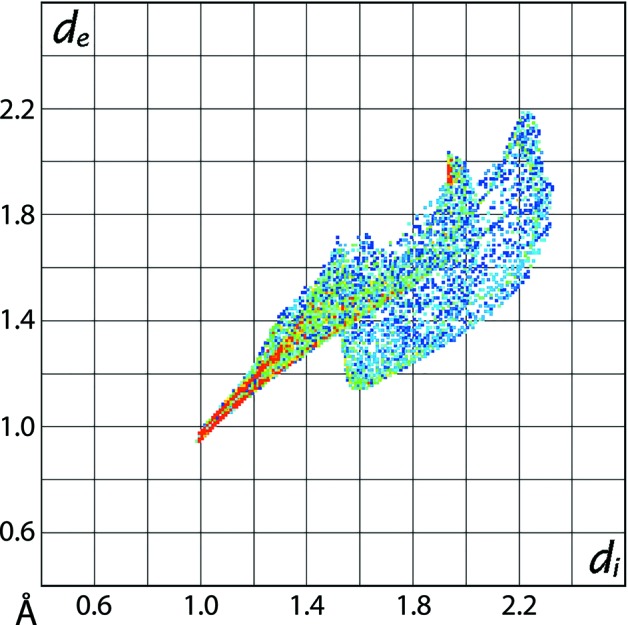
The two-dimensional fingerprint plot taking into account only the Hirshfeld surface calculated about the copper(II) atom.

The Hirshfeld surfaces also provide an insight into the distortion in the coordination geometry formed by the N_2_O_4_ donor set about the copper(II) centre in the complex mol­ecule. This is performed by considering the Hirshfeld surface about the metal centre alone (Pinto *et al.*, 2019 ▸). The distortion in the coordination geometry is observed on the Hirshfeld surface mapped with the shape-index property as the bright-orange patches of irregular shape covering a major region for the Cu—O bonds in Fig. 8 ▸(*a*) and the small orange regions on the surface relatively far from the Cu—N bonds in Fig. 8 ▸(*b*). The different curvature of the Hirshfeld surfaces coordinated by the N_2_O_4_ donor set in Figs. 8 ▸(*c*) and 8(*d*) also support this observation. The Cu—O and Cu—N bonds are rationalized in the two-dimensional fingerprint plot taking into account only the Hirshfeld surface for the copper atom shown in Fig. 9 ▸. The distribution of points in the fingerprint plot through the pair of aligned red points at different inclinations from *d*_e_ + *d*_i_ ∼ 2.0 Å for the Cu—N bonds (upper region) and the Cu—O bonds (lower region) are indicative of the distorted geometry (Pinto *et al.*, 2019 ▸).

**Figure 10:**
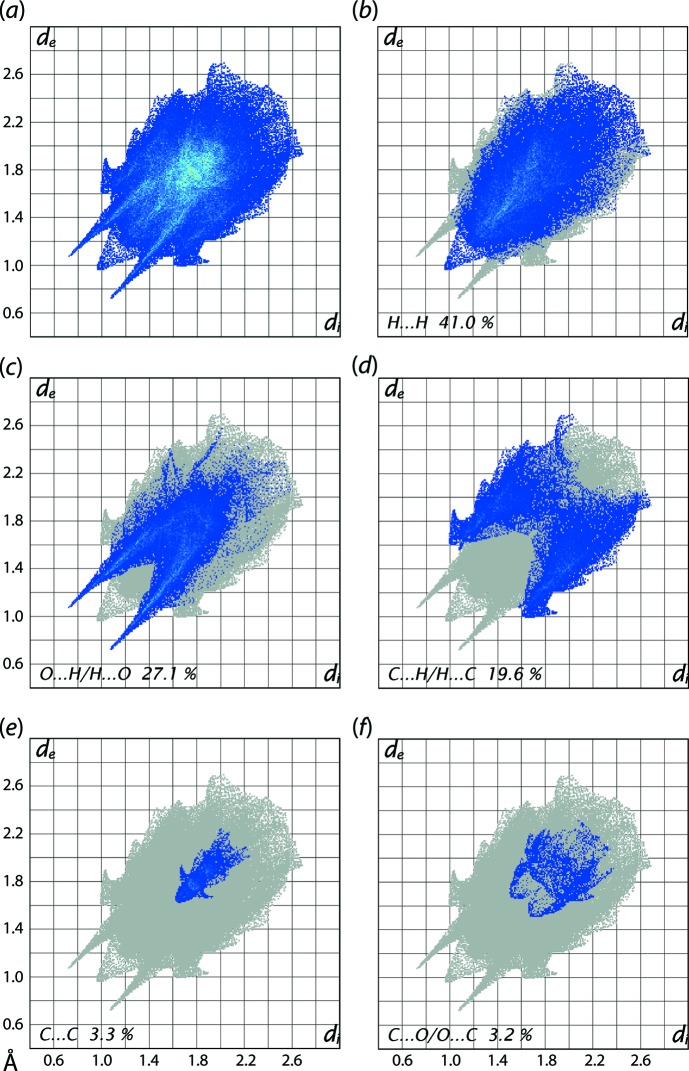
(*a*) A comparison of the full two-dimensional fingerprint plot for (I) and those delineated into (*b*) H⋯H, (*c*) O⋯H/H⋯O, (*d*) C⋯H/H⋯C, (*e*) C⋯C and (*f*) C⋯O/O⋯C contacts.

The overall two-dimensional fingerprint plot for (I), *i.e.* the entire asymmetric unit, Fig. 10 ▸(*a*), and those delineated into H⋯H, O⋯H/H⋯O, C⋯H/H⋯C, C⋯C and C⋯O/O⋯C contacts are illustrated in Figs. 10 ▸(*b*)–(*f*), respectively. The percentage contribution from different inter­atomic contacts to the Hirshfeld surfaces of the complex mol­ecule and for overall (I) are summarized in Table 4 ▸. The presence of water mol­ecules in the crystal of (I) increases the percentage contribution from O⋯H/H⋯O contacts (Table 4 ▸) to the Hirshfeld surface of the asymmetric unit compared with the complex mol­ecule alone. This results in slight decreases in the percentage contributions from other inter­atomic contacts for (I) (Table 4 ▸). A single conical tip at *d*_e_ + *d*_i_ ∼ 1.9 Å in the fingerprint plot delineated into H⋯H contacts shown in Fig. 10 ▸(*b*) is the result of the involvement of the H12 atom in a short inter­atomic H⋯H contact, Table 3 ▸. The points due to short inter­atomic contacts between amine hydrogen-H3N and water hydrogen atoms, H1*W* and H2*W*, Table 3 ▸, are merged within the plot. Although the mol­ecular packing of (I) is influenced by several inter­molecular O—H⋯O and C—H⋯O inter­actions, the presence of a pair of long spikes at *d*_e_ + *d*_i_ ∼ 1.8 Å in the plot delineated into O⋯H/H⋯O contacts, Fig. 10 ▸(*c*), arise from the N—H⋯O hydrogen bond, while the merged points correspond to other inter­actions at greater inter­atomic distances. The significant contribution from inter­atomic C⋯H/H⋯C contacts (Table 4 ▸) to the Hirshfeld surface of (I) reflect the combined influence of inter­molecular C—H⋯π inter­actions (Table 2 ▸) and the short inter­atomic C⋯H/H⋯C contacts, summarized in Table 3 ▸, and viewed as the distribution of points in the form of characteristic wings in Fig. 10 ▸(*d*). The presence of short inter­atomic C⋯C contacts are evident as the points near a rocket shape tip at *d*_e_ + *d*_i_ ∼ 3.2 Å in the respective delineated fingerprint plot, Fig. 10 ▸(*e*), while the points corresponding π–π stacking between the imidazole and nitro­benzene rings are distributed about *d*_e_ = *d*_i_ = 1.7 Å in the plot. The small, *i.e*. 2.7%, contribution from C⋯N/N⋯C contacts to the surface is also due to these π–π stacking inter­actions (delineated plot not shown). The contribution of 3.2% from C⋯O/O⋯C contacts is due to the presence of short inter­atomic contacts involving nitro-O atoms, Table 2 ▸, and are apparent as the pair of parabolic tips at *d*_e_ + *d*_i_ ∼ 3.2 Å in the delineated plot of Fig. 10 ▸(*f*). The contribution from other inter­atomic contacts to the surface summarized in Table 4 ▸ have negligible influence on the mol­ecular packing.

## Database survey   

There are five crystal structures of copper complexes with related 2-(4,5-diphenyl-1*H*-imidazol-2-yl)phenolate ligands in the literature [Cambridge Structural Database (CSD): Groom *et al.*, 2016 ▸]. The first of these is the 4-bromo derivative of (I), isolated as a di­methyl­formamide solvate [(II); CSD refcode YUKSOO] (Parween *et al.*, 2015 ▸). The remaining four structures are 2,4-(*t*-Bu)_2_-phenolate derivatives, three of which are copper(II) complexes and the other, a copper(III) complex. Three of these four species have no additional substitution (Benisvy *et al.*, 2003 ▸). One was isolated as a methanol tris­olvate [(III); JADZUK], another as a di­methyl­formamide tetra­solvate [(IV); NEPLAV01] and the third an oxidized species, *i.e*. a copper(III) complex, was isolated as a tetra­fluoro­borate salt/di­chloro­methane disolvate [(V); NEP­LEZ01]; complex (IV) has crystallographic twofold symmetry. The final structure, a copper(II) complex (Benisvy *et al.*, 2006 ▸), has additional 4-meth­oxy­phenyl substituents on the imidazol-2-yl rings and was isolated as a methanol disolvate [(VI); JEBRUE]. The common feature of all the structures is the ‘*cis*’-N_2_O_2_ set but the coordination geometries are highly distorted, as seen in the sequence of τ_4_ values for (I)–(VI) of 0.48, 0.53, 0.44, 0.37, 0.47 and 0.35, respectively.

## Synthesis and crystallization   

In a typical procedure, benzil (0.3 g, 1 mmol), ammonium acetate (0.19 g, 2.5 mmol), 2-hy­droxy-5-nitro­benzalaldehyde (0.167 g, 1 mmol) and copper(II) borate (0.218 mg, 1 mmol) were ground in an agate mortar with a pestle. To this mixture, about 1.5 g of dried silica gel (column chromatography, 60–120 mesh) was added and the reaction mixture was ground again for 30 min. The whole reaction mixture was then transferred to a 100 ml round-bottomed flask and heated at 130 °C with constant stirring for 4 h. The reaction mixture was then extracted with dry acetone and dried over MgSO_4_. After a few days, a dark-brown solid was obtained. The product was recrystallized from dry di­methyl­formamide and, after 5 d, light-blue crystals of (I) were obtained (yield 60%; m.p. > 300 °C).

## Refinement   

Crystal data, data collection and structure refinement details are summarized in Table 5 ▸. Carbon-bound H-atoms were placed in calculated positions (C—H = 0.95 Å) and were included in the refinement in the riding-model approximation, with *U*_iso_(H) values set at 1.2*U*_eq_(C). The O- and N-bound H atoms were located in a difference Fourier map but were refined with distance restraints of O—H = 0.84 ± 0.01 Å and N—H = 0.88 ± 0.01 Å, respectively, and with *U*_iso_(H) set at 1.5*U*_eq_(O) or 1.2*U*_eq_(N).

## Supplementary Material

Crystal structure: contains datablock(s) . DOI: 10.1107/S2056989019013720/hb7859sup1.cif

Structure factors: contains datablock(s) I. DOI: 10.1107/S2056989019013720/hb7859Isup2.hkl

CCDC references: 1958158, 1958158

Additional supporting information:  crystallographic information; 3D view; checkCIF report

## Figures and Tables

**Table 1:** Selected geometric parameters (Å, °)

Cu—O1	1.9291 (17)	Cu—N1	1.9586 (19)
Cu—O2	1.9304 (17)	Cu—N2	1.957 (2)
			
O1—Cu—O2	89.36 (7)	O2—Cu—N1	144.41 (8)
O1—Cu—N2	147.34 (8)	O2—Cu—N2	93.56 (7)
O1—Cu—N1	92.83 (8)	N1—Cu—N2	103.14 (8)

**Table 2:** Hydrogen-bond geometry (Å, °) *Cg*1 is the ring centroid of the C16–C21 ring.

*D*—H⋯*A*	*D*—H	H⋯*A*	*D*⋯*A*	*D*—H⋯*A*
N3—H3*N*⋯O1*W* ^i^	0.89 (2)	1.91 (2)	2.790 (3)	173 (3)
N5—H5*N*⋯O2*W*	0.88 (2)	1.95 (2)	2.822 (3)	172 (3)
O1*W*—H1*W*⋯O2	0.85 (2)	1.92 (2)	2.745 (2)	164 (2)
O1*W*—H2*W*⋯O2*W* ^ii^	0.85 (2)	2.21 (2)	2.868 (2)	134 (2)
O2*W*—H3*W*⋯O1^ii^	0.84 (2)	2.01 (2)	2.841 (2)	172 (2)
O2*W*—H4*W*⋯O3^iii^	0.84 (2)	2.27 (2)	2.938 (2)	136 (2)
C3—H3⋯O1*W* ^i^	0.95	2.57	3.435 (3)	151
C33—H33⋯O5^iv^	0.95	2.48	3.345 (3)	151
C5—H5⋯O6^v^	0.95	2.50	3.361 (3)	151
C34—H34⋯*Cg*1^vi^	0.95	2.49	3.426 (3)	168

Symmetry codes: (i) 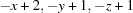; (ii) 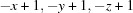; (iii) 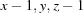; (iv) 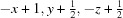; (v) 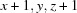; (vi) 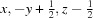.

**Table 3:** Summary of short inter­atomic contacts (Å) in (I)^*a*^

Contact	Distance	Symmetry operation
H12⋯H12	1.92	−*x* + 1, −*y* + 1, −*z* + 1
H1*W*⋯H3N	2.22	−*x* + 2, −*y* + 1, −*z* + 1
H2*W*⋯H3N	2.26	−*x* + 2, −*y* + 1, −*z* + 1
O4⋯H40	2.54	*x* + 1, −*y* + 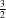 , *z* + 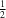
C1⋯H3*W*	2.74	−*x* + 1, −*y* + 1, −*z* + 1
C6⋯O6	3.206 (3)	−*x* + 1, −*y* + 1, −*z* + 1
C12⋯H12	2.55	−*x* + 1, −*y* + 1, −*z*
C13⋯C25	3.347 (3)	−*x* + 1, −*y* + 1, −*z*
C14⋯O5	3.197 (3)	−*x* + 1, −*y* + 1, −*z*
H17⋯O6	2.55	−*x* + 1, −*y* + 1, −*z*
C19⋯H34	2.68	*x*, −*y* + 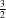 , *z* − 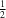
C20⋯H34	2.60	*x*, −*y* + 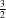 , *z* − 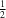
C21⋯H34	2.67	*x*, −*y* + 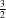 , *z* − 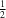
C21⋯H2*W*	2.64	−*x* + 2, −*y* + 1, −*z* + 1
C21⋯O1*W*	3.161 (3)	−*x* + 2, −*y* + 1, −*z* + 1
C22⋯C28	3.267 (3)	−*x* + 1, −*y* + 1, −*z* + 1
C36⋯O5	3.146 (3)	−*x* + 1, −*y* + 1, −*z* + 1
H36⋯O5	2.49	−*x* + 1, −*y* + 1, −*z* + 1
C41⋯H20	2.76	−*x* + 1, *y*, *z*

Notes: (*a*) the inter­atomic distances are calculated in *CrystalExplorer17* (Turner *et al.*, 2017 ▸), whereby the *X*—H bond lengths are adjusted to their neutron values.

**Table 4:** Percentage contributions of inter­atomic contacts to the Hirshfeld surface for the complex mol­ecule in (I) and overall (I)

Contact	Percentage contribution	
	complex mol­ecule	(I)
H⋯H	41.3	41.0
O⋯H/H⋯O	25.6	27.1
C⋯H/H⋯C	19.8	19.6
C⋯C	3.5	3.3
C⋯O/O⋯C	3.4	3.2
C⋯N/N⋯C	2.8	2.7
N⋯H/H⋯N	2.2	2.1
O⋯O	0.6	0.5
N⋯O/O⋯N	0.2	0.2
Cu⋯O/O⋯Cu	0.0	0.3
Cu⋯C/C⋯Cu	0.3	0.0

**Table 5:** Experimental details

Crystal data	
Chemical formula	[Cu(C_21_H_14_N_3_O_3_)_2_]·2H_2_O
*M*_r_	812.27
Crystal system, space group	Monoclinic, *P*2_1_/*c*
Temperature (K)	100
*a*, *b*, *c* (Å)	13.2752 (2), 25.1602 (4), 11.1166 (2)
β (°)	104.256 (1)
*V* (Å^3^)	3598.68 (10)
*Z*	4
Radiation type	Cu *K*α
μ (mm^−1^)	1.42
Crystal size (mm)	0.14 × 0.11 × 0.07

Data collection	
Diffractometer	XtaLAB Synergy, Dualflex, AtlasS2
Absorption correction	Gaussian (*CrysAlis PRO*; Rigaku OD, 2018 ▸)
*T*_min_, *T*_max_	0.757, 1.000
No. of measured, independent and observed [*I* > 2σ(*I*)] reflections	46023, 7490, 6420
*R*_int_	0.058
(sin θ/λ)_max_ (Å^−1^)	0.631

Refinement	
*R*[*F* ^2^ > 2σ(*F* ^2^)], *wR*(*F* ^2^), *S*	0.047, 0.128, 1.05
No. of reflections	7490
No. of parameters	532
No. of restraints	8
H-atom treatment	H atoms treated by a mixture of independent and constrained refinement
Δρ_max_, Δρ_min_ (e Å^−3^)	0.61, −0.74

Computer programs: *CrysAlis PRO* (Rigaku OD, 2018 ▸), *SHELXS* (Sheldrick, 2015*a* ▸), *SHELXL2014* (Sheldrick, 2015*b* ▸), *ORTEP-3 for Windows* (Farrugia, 2012 ▸), *DIAMOND* (Brandenburg, 2006 ▸) and *publCIF* (Westrip, 2010 ▸).

## supplementary crystallographic information

### Crystal data

**Table:**

[Cu(C_21_H_14_N_3_O_3_)_2_]·2H_2_O	*F*(000) = 1676
*M**_r_* = 812.27	*D*_x_ = 1.499 Mg m^−^^3^
Monoclinic, *P*2_1_/*c*	Cu *K*α radiation, λ = 1.54178 Å
*a* = 13.2752 (2) Å	Cell parameters from 17322 reflections
*b* = 25.1602 (4) Å	θ = 3.4–75.1°
*c* = 11.1166 (2) Å	µ = 1.42 mm^−^^1^
β = 104.256 (1)°	*T* = 100 K
*V* = 3598.68 (10) Å^3^	Block, light-blue
*Z* = 4	0.14 × 0.11 × 0.07 mm

### Data collection

**Table:**

XtaLAB Synergy, Dualflex, AtlasS2 diffractometer	7490 independent reflections
Radiation source: micro-focus sealed X-ray tube	6420 reflections with *I* > 2σ(*I*)
Detector resolution: 5.2558 pixels mm^-1^	*R*_int_ = 0.058
ω scans	θ_max_ = 76.5°, θ_min_ = 3.4°
Absorption correction: gaussian (CrysAlis PRO; Rigaku OD, 2018)	*h* = −16→15
*T*_min_ = 0.757, *T*_max_ = 1.000	*k* = −31→18
46023 measured reflections	*l* = −14→13

### Refinement

**Table:**

Refinement on *F*^2^	Primary atom site location: structure-invariant direct methods
Least-squares matrix: full	Secondary atom site location: difference Fourier map
*R*[*F*^2^ > 2σ(*F*^2^)] = 0.047	Hydrogen site location: mixed
*wR*(*F*^2^) = 0.128	H atoms treated by a mixture of independent and constrained refinement
*S* = 1.05	*w* = 1/[σ^2^(*F*_o_^2^) + (0.0612*P*)^2^ + 4.3316*P*] where *P* = (*F*_o_^2^ + 2*F*_c_^2^)/3
7490 reflections	(Δ/σ)_max_ = 0.001
532 parameters	Δρ_max_ = 0.61 e Å^−^^3^
8 restraints	Δρ_min_ = −0.74 e Å^−^^3^

### Special details

**Table:**

Geometry. All esds (except the esd in the dihedral angle between two l.s. planes) are estimated using the full covariance matrix. The cell esds are taken into account individually in the estimation of esds in distances, angles and torsion angles; correlations between esds in cell parameters are only used when they are defined by crystal symmetry. An approximate (isotropic) treatment of cell esds is used for estimating esds involving l.s. planes.

### Fractional atomic coordinates and isotropic or equivalent isotropic displacement parameters (Å^2^)

**Table:**

	*x*	*y*	*z*	*U*_iso_*/*U*_eq_	
Cu	0.74255 (2)	0.52876 (2)	0.48001 (3)	0.01988 (11)	
O1	0.83947 (13)	0.50064 (7)	0.62462 (16)	0.0252 (4)	
O2	0.66268 (12)	0.46402 (7)	0.46842 (16)	0.0230 (3)	
O3	1.24824 (14)	0.57484 (8)	0.99386 (16)	0.0330 (4)	
O4	1.27722 (17)	0.60469 (10)	0.82272 (19)	0.0473 (6)	
O5	0.24188 (14)	0.37240 (7)	0.15743 (17)	0.0293 (4)	
O6	0.22116 (14)	0.45563 (7)	0.10696 (17)	0.0306 (4)	
N1	0.85211 (14)	0.56519 (8)	0.42190 (18)	0.0205 (4)	
N2	0.62006 (14)	0.57293 (8)	0.41429 (17)	0.0195 (4)	
N3	1.00287 (15)	0.60284 (8)	0.43184 (18)	0.0208 (4)	
H3N	1.0706 (14)	0.6096 (12)	0.456 (3)	0.025*	
N4	1.22194 (17)	0.58167 (9)	0.8805 (2)	0.0288 (5)	
N5	0.46035 (15)	0.59145 (8)	0.31342 (18)	0.0209 (4)	
H5N	0.3926 (14)	0.5882 (12)	0.288 (3)	0.025*	
N6	0.27280 (15)	0.41869 (8)	0.16466 (18)	0.0232 (4)	
C1	0.93103 (18)	0.52034 (9)	0.6799 (2)	0.0217 (5)	
C2	0.98664 (18)	0.55672 (9)	0.6228 (2)	0.0206 (4)	
C3	1.08255 (18)	0.57635 (10)	0.6906 (2)	0.0239 (5)	
H3	1.1200	0.6008	0.6532	0.029*	
C4	1.12307 (18)	0.56018 (10)	0.8122 (2)	0.0232 (5)	
C5	1.07118 (19)	0.52365 (10)	0.8690 (2)	0.0246 (5)	
H5	1.1001	0.5124	0.9519	0.030*	
C6	0.97736 (18)	0.50408 (10)	0.8029 (2)	0.0246 (5)	
H6	0.9423	0.4787	0.8411	0.029*	
C7	0.94688 (17)	0.57399 (9)	0.4945 (2)	0.0201 (4)	
C8	0.84752 (17)	0.58953 (9)	0.3082 (2)	0.0202 (4)	
C9	0.94244 (17)	0.61300 (9)	0.3143 (2)	0.0205 (4)	
C10	0.75336 (18)	0.58906 (10)	0.2072 (2)	0.0224 (5)	
C11	0.69242 (19)	0.54320 (11)	0.1822 (2)	0.0250 (5)	
H11	0.7152	0.5115	0.2269	0.030*	
C12	0.5990 (2)	0.54366 (12)	0.0927 (2)	0.0320 (6)	
H12	0.5578	0.5124	0.0772	0.038*	
C13	0.5652 (2)	0.58977 (13)	0.0253 (2)	0.0326 (6)	
H13	0.5006	0.5903	−0.0351	0.039*	
C14	0.6269 (2)	0.63488 (12)	0.0476 (2)	0.0299 (6)	
H14	0.6051	0.6662	0.0005	0.036*	
C15	0.71990 (19)	0.63494 (11)	0.1373 (2)	0.0254 (5)	
H15	0.7612	0.6662	0.1517	0.031*	
C16	0.98685 (17)	0.64082 (10)	0.2229 (2)	0.0217 (5)	
C17	0.96256 (18)	0.62504 (10)	0.0983 (2)	0.0239 (5)	
H17	0.9122	0.5981	0.0701	0.029*	
C18	1.0124 (2)	0.64895 (11)	0.0159 (2)	0.0291 (5)	
H18	0.9954	0.6383	−0.0687	0.035*	
C19	1.0863 (2)	0.68803 (12)	0.0558 (3)	0.0323 (6)	
H19	1.1218	0.7032	−0.0003	0.039*	
C20	1.1084 (2)	0.70494 (11)	0.1786 (3)	0.0311 (6)	
H20	1.1578	0.7324	0.2060	0.037*	
C21	1.05857 (18)	0.68174 (10)	0.2611 (2)	0.0247 (5)	
H21	1.0734	0.6938	0.3446	0.030*	
C22	0.56875 (17)	0.45490 (9)	0.3982 (2)	0.0200 (4)	
C23	0.50083 (18)	0.49614 (9)	0.3407 (2)	0.0204 (4)	
C24	0.40437 (18)	0.48271 (10)	0.2621 (2)	0.0214 (5)	
H24	0.3598	0.5099	0.2198	0.026*	
C25	0.37391 (17)	0.43039 (10)	0.2459 (2)	0.0209 (5)	
C26	0.43829 (18)	0.38910 (10)	0.3045 (2)	0.0225 (5)	
H26	0.4163	0.3531	0.2930	0.027*	
C27	0.53397 (18)	0.40181 (10)	0.3791 (2)	0.0230 (5)	
H27	0.5782	0.3740	0.4191	0.028*	
C28	0.52750 (17)	0.55192 (9)	0.3586 (2)	0.0195 (4)	
C29	0.61089 (17)	0.62771 (9)	0.4033 (2)	0.0192 (4)	
C30	0.51046 (18)	0.63939 (9)	0.3401 (2)	0.0212 (5)	
C31	0.69704 (18)	0.66474 (9)	0.4539 (2)	0.0214 (5)	
C32	0.70930 (19)	0.71076 (10)	0.3893 (2)	0.0255 (5)	
H32	0.6635	0.7179	0.3107	0.031*	
C33	0.7886 (2)	0.74629 (11)	0.4399 (3)	0.0318 (6)	
H33	0.7971	0.7777	0.3959	0.038*	
C34	0.8553 (2)	0.73593 (11)	0.5545 (3)	0.0340 (6)	
H34	0.9089	0.7605	0.5892	0.041*	
C35	0.8443 (2)	0.69010 (11)	0.6187 (3)	0.0316 (6)	
H35	0.8906	0.6830	0.6968	0.038*	
C36	0.76519 (19)	0.65455 (10)	0.5684 (2)	0.0245 (5)	
H36	0.7575	0.6230	0.6124	0.029*	
C37	0.45587 (18)	0.68949 (10)	0.2984 (2)	0.0228 (5)	
C38	0.46133 (19)	0.73260 (10)	0.3798 (2)	0.0262 (5)	
H38	0.5002	0.7296	0.4635	0.031*	
C39	0.4098 (2)	0.77969 (10)	0.3378 (3)	0.0289 (5)	
H39	0.4135	0.8088	0.3933	0.035*	
C40	0.35291 (19)	0.78459 (10)	0.2151 (3)	0.0284 (5)	
H40	0.3180	0.8169	0.1868	0.034*	
C41	0.34751 (19)	0.74196 (11)	0.1343 (2)	0.0279 (5)	
H41	0.3090	0.7452	0.0504	0.034*	
C42	0.39843 (19)	0.69450 (10)	0.1760 (2)	0.0255 (5)	
H42	0.3939	0.6654	0.1205	0.031*	
O1W	0.78491 (13)	0.37472 (7)	0.51477 (16)	0.0256 (4)	
H1W	0.753 (2)	0.4034 (8)	0.489 (2)	0.038*	
H2W	0.778 (2)	0.3679 (11)	0.5869 (15)	0.038*	
O2W	0.24174 (13)	0.58295 (7)	0.25549 (16)	0.0255 (4)	
H3W	0.221 (2)	0.5561 (9)	0.287 (2)	0.038*	
H4W	0.210 (2)	0.5863 (12)	0.1806 (12)	0.038*	

### Atomic displacement parameters (Å^2^)

**Table:**

	*U*^11^	*U*^22^	*U*^33^	*U*^12^	*U*^13^	*U*^23^
Cu	0.01593 (17)	0.02201 (19)	0.02094 (18)	−0.00086 (12)	0.00306 (13)	0.00226 (13)
O1	0.0202 (8)	0.0287 (9)	0.0257 (8)	−0.0045 (7)	0.0035 (7)	0.0039 (7)
O2	0.0167 (7)	0.0238 (8)	0.0271 (9)	−0.0004 (6)	0.0026 (6)	0.0021 (7)
O3	0.0316 (10)	0.0436 (11)	0.0194 (8)	−0.0056 (8)	−0.0023 (7)	0.0008 (8)
O4	0.0359 (11)	0.0665 (15)	0.0329 (11)	−0.0253 (10)	−0.0043 (9)	0.0158 (10)
O5	0.0302 (9)	0.0269 (9)	0.0290 (9)	−0.0097 (7)	0.0040 (7)	−0.0014 (7)
O6	0.0245 (9)	0.0302 (10)	0.0315 (9)	0.0023 (7)	−0.0037 (7)	0.0009 (8)
N1	0.0161 (9)	0.0247 (10)	0.0195 (9)	−0.0003 (7)	0.0024 (7)	0.0011 (7)
N2	0.0175 (9)	0.0212 (9)	0.0199 (9)	−0.0027 (7)	0.0046 (7)	−0.0009 (7)
N3	0.0152 (9)	0.0259 (10)	0.0201 (9)	0.0000 (7)	0.0025 (7)	0.0026 (8)
N4	0.0279 (11)	0.0312 (11)	0.0243 (10)	−0.0061 (9)	0.0007 (8)	0.0025 (9)
N5	0.0171 (9)	0.0217 (10)	0.0233 (10)	−0.0004 (7)	0.0039 (8)	0.0002 (8)
N6	0.0229 (10)	0.0268 (10)	0.0195 (9)	−0.0028 (8)	0.0046 (8)	−0.0019 (8)
C1	0.0182 (10)	0.0230 (11)	0.0233 (11)	0.0010 (9)	0.0036 (9)	0.0015 (9)
C2	0.0200 (11)	0.0219 (11)	0.0194 (11)	0.0009 (9)	0.0041 (9)	0.0003 (9)
C3	0.0217 (11)	0.0261 (12)	0.0229 (11)	−0.0026 (9)	0.0037 (9)	0.0020 (9)
C4	0.0199 (11)	0.0268 (12)	0.0207 (11)	−0.0012 (9)	0.0009 (9)	−0.0013 (9)
C5	0.0243 (12)	0.0265 (12)	0.0222 (11)	0.0011 (9)	0.0044 (9)	0.0021 (9)
C6	0.0227 (11)	0.0274 (12)	0.0234 (11)	−0.0011 (9)	0.0053 (9)	0.0039 (9)
C7	0.0152 (10)	0.0231 (11)	0.0218 (11)	−0.0018 (8)	0.0042 (8)	0.0006 (9)
C8	0.0188 (11)	0.0244 (11)	0.0171 (10)	−0.0009 (9)	0.0040 (8)	0.0010 (8)
C9	0.0171 (10)	0.0242 (12)	0.0190 (11)	0.0008 (9)	0.0021 (8)	0.0015 (9)
C10	0.0183 (11)	0.0293 (12)	0.0199 (11)	0.0002 (9)	0.0052 (9)	−0.0009 (9)
C11	0.0221 (11)	0.0329 (13)	0.0194 (11)	−0.0035 (10)	0.0041 (9)	−0.0004 (9)
C12	0.0261 (13)	0.0445 (16)	0.0248 (12)	−0.0091 (11)	0.0054 (10)	−0.0060 (11)
C13	0.0215 (12)	0.0552 (18)	0.0192 (11)	0.0012 (11)	0.0012 (9)	−0.0006 (11)
C14	0.0259 (12)	0.0422 (15)	0.0212 (11)	0.0086 (11)	0.0053 (10)	0.0054 (10)
C15	0.0232 (12)	0.0321 (13)	0.0218 (11)	0.0012 (10)	0.0070 (9)	0.0023 (10)
C16	0.0174 (10)	0.0251 (12)	0.0225 (11)	0.0018 (9)	0.0048 (9)	0.0034 (9)
C17	0.0207 (11)	0.0288 (12)	0.0218 (11)	0.0017 (9)	0.0045 (9)	0.0029 (9)
C18	0.0268 (12)	0.0362 (14)	0.0251 (12)	0.0071 (10)	0.0083 (10)	0.0061 (10)
C19	0.0254 (12)	0.0389 (15)	0.0357 (14)	0.0031 (11)	0.0133 (11)	0.0113 (11)
C20	0.0220 (12)	0.0316 (14)	0.0395 (14)	−0.0026 (10)	0.0074 (11)	0.0086 (11)
C21	0.0193 (11)	0.0271 (12)	0.0255 (12)	−0.0004 (9)	0.0015 (9)	0.0046 (9)
C22	0.0176 (10)	0.0242 (11)	0.0190 (10)	−0.0023 (9)	0.0058 (8)	−0.0010 (9)
C23	0.0212 (11)	0.0209 (11)	0.0201 (10)	−0.0008 (9)	0.0067 (9)	0.0002 (9)
C24	0.0221 (11)	0.0235 (11)	0.0191 (11)	0.0006 (9)	0.0060 (9)	0.0012 (9)
C25	0.0178 (11)	0.0254 (12)	0.0195 (10)	−0.0025 (9)	0.0047 (9)	−0.0020 (9)
C26	0.0219 (11)	0.0229 (12)	0.0232 (11)	−0.0025 (9)	0.0061 (9)	−0.0018 (9)
C27	0.0224 (11)	0.0225 (12)	0.0246 (11)	0.0016 (9)	0.0066 (9)	0.0010 (9)
C28	0.0169 (10)	0.0242 (12)	0.0184 (10)	−0.0001 (8)	0.0059 (8)	0.0005 (8)
C29	0.0174 (10)	0.0218 (11)	0.0178 (10)	−0.0012 (8)	0.0032 (8)	−0.0004 (8)
C30	0.0206 (11)	0.0219 (11)	0.0217 (11)	−0.0018 (9)	0.0063 (9)	−0.0008 (9)
C31	0.0195 (11)	0.0221 (11)	0.0237 (11)	−0.0018 (9)	0.0074 (9)	−0.0023 (9)
C32	0.0248 (12)	0.0227 (12)	0.0290 (12)	0.0007 (9)	0.0065 (10)	0.0003 (10)
C33	0.0287 (13)	0.0234 (12)	0.0439 (15)	−0.0040 (10)	0.0103 (11)	0.0004 (11)
C34	0.0278 (13)	0.0293 (14)	0.0431 (16)	−0.0086 (10)	0.0052 (12)	−0.0086 (11)
C35	0.0261 (13)	0.0351 (14)	0.0307 (13)	−0.0042 (10)	0.0017 (10)	−0.0066 (11)
C36	0.0228 (11)	0.0279 (12)	0.0230 (11)	−0.0015 (9)	0.0060 (9)	−0.0011 (9)
C37	0.0205 (11)	0.0229 (12)	0.0260 (12)	−0.0004 (9)	0.0078 (9)	0.0007 (9)
C38	0.0252 (12)	0.0245 (12)	0.0285 (12)	−0.0008 (9)	0.0060 (10)	−0.0016 (10)
C39	0.0254 (12)	0.0249 (12)	0.0373 (14)	0.0010 (10)	0.0098 (11)	−0.0016 (10)
C40	0.0232 (12)	0.0261 (13)	0.0379 (14)	0.0015 (10)	0.0115 (10)	0.0064 (10)
C41	0.0238 (12)	0.0322 (13)	0.0279 (13)	0.0021 (10)	0.0065 (10)	0.0060 (10)
C42	0.0242 (12)	0.0256 (12)	0.0264 (12)	0.0008 (9)	0.0059 (10)	0.0019 (9)
O1W	0.0210 (8)	0.0285 (9)	0.0263 (9)	0.0014 (7)	0.0038 (7)	0.0020 (7)
O2W	0.0213 (8)	0.0295 (9)	0.0246 (8)	−0.0014 (7)	0.0035 (7)	0.0039 (7)

### Geometric parameters (Å, º)

**Table:**

Cu—O1	1.9291 (17)	C17—C18	1.392 (3)
Cu—O2	1.9304 (17)	C17—H17	0.9500
Cu—N1	1.9586 (19)	C18—C19	1.383 (4)
Cu—N2	1.957 (2)	C18—H18	0.9500
O1—C1	1.316 (3)	C19—C20	1.391 (4)
O2—C22	1.318 (3)	C19—H19	0.9500
O3—N4	1.234 (3)	C20—C21	1.384 (4)
O4—N4	1.232 (3)	C20—H20	0.9500
O5—N6	1.231 (3)	C21—H21	0.9500
O6—N6	1.236 (3)	C22—C27	1.412 (3)
N1—C7	1.335 (3)	C22—C23	1.420 (3)
N1—C8	1.392 (3)	C23—C24	1.402 (3)
N2—C28	1.341 (3)	C23—C28	1.449 (3)
N2—C29	1.386 (3)	C24—C25	1.376 (3)
N3—C7	1.349 (3)	C24—H24	0.9500
N3—C9	1.378 (3)	C25—C26	1.400 (3)
N3—H3N	0.889 (17)	C26—C27	1.373 (3)
N4—C4	1.449 (3)	C26—H26	0.9500
N5—C28	1.347 (3)	C27—H27	0.9500
N5—C30	1.374 (3)	C29—C30	1.377 (3)
N5—H5N	0.877 (17)	C29—C31	1.475 (3)
N6—C25	1.452 (3)	C30—C37	1.471 (3)
C1—C6	1.415 (3)	C31—C36	1.392 (3)
C1—C2	1.420 (3)	C31—C32	1.393 (3)
C2—C3	1.400 (3)	C32—C33	1.390 (4)
C2—C7	1.459 (3)	C32—H32	0.9500
C3—C4	1.387 (3)	C33—C34	1.385 (4)
C3—H3	0.9500	C33—H33	0.9500
C4—C5	1.390 (3)	C34—C35	1.382 (4)
C5—C6	1.372 (3)	C34—H34	0.9500
C5—H5	0.9500	C35—C36	1.388 (3)
C6—H6	0.9500	C35—H35	0.9500
C8—C9	1.378 (3)	C36—H36	0.9500
C8—C10	1.460 (3)	C37—C42	1.392 (3)
C9—C16	1.471 (3)	C37—C38	1.403 (3)
C10—C11	1.397 (3)	C38—C39	1.390 (4)
C10—C15	1.401 (4)	C38—H38	0.9500
C11—C12	1.385 (4)	C39—C40	1.393 (4)
C11—H11	0.9500	C39—H39	0.9500
C12—C13	1.394 (4)	C40—C41	1.390 (4)
C12—H12	0.9500	C40—H40	0.9500
C13—C14	1.386 (4)	C41—C42	1.394 (4)
C13—H13	0.9500	C41—H41	0.9500
C14—C15	1.383 (3)	C42—H42	0.9500
C14—H14	0.9500	O1W—H1W	0.851 (10)
C15—H15	0.9500	O1W—H2W	0.847 (10)
C16—C21	1.396 (3)	O2W—H3W	0.843 (10)
C16—C17	1.400 (3)	O2W—H4W	0.840 (10)
			
O1—Cu—O2	89.36 (7)	C19—C18—C17	120.7 (2)
O1—Cu—N2	147.34 (8)	C19—C18—H18	119.6
O1—Cu—N1	92.83 (8)	C17—C18—H18	119.6
O2—Cu—N1	144.41 (8)	C18—C19—C20	119.6 (2)
O2—Cu—N2	93.56 (7)	C18—C19—H19	120.2
N1—Cu—N2	103.14 (8)	C20—C19—H19	120.2
C1—O1—Cu	126.91 (15)	C21—C20—C19	120.1 (2)
C22—O2—Cu	127.61 (15)	C21—C20—H20	120.0
C7—N1—C8	107.45 (19)	C19—C20—H20	120.0
C7—N1—Cu	122.96 (16)	C20—C21—C16	120.8 (2)
C8—N1—Cu	129.44 (15)	C20—C21—H21	119.6
C28—N2—C29	107.50 (19)	C16—C21—H21	119.6
C28—N2—Cu	122.09 (16)	O2—C22—C27	118.7 (2)
C29—N2—Cu	130.13 (15)	O2—C22—C23	122.9 (2)
C7—N3—C9	108.85 (19)	C27—C22—C23	118.4 (2)
C7—N3—H3N	126.5 (19)	C24—C23—C22	119.1 (2)
C9—N3—H3N	123.8 (19)	C24—C23—C28	118.3 (2)
O4—N4—O3	122.9 (2)	C22—C23—C28	122.6 (2)
O4—N4—C4	118.8 (2)	C25—C24—C23	120.4 (2)
O3—N4—C4	118.4 (2)	C25—C24—H24	119.8
C28—N5—C30	109.1 (2)	C23—C24—H24	119.8
C28—N5—H5N	126 (2)	C24—C25—C26	121.5 (2)
C30—N5—H5N	124 (2)	C24—C25—N6	118.2 (2)
O5—N6—O6	123.0 (2)	C26—C25—N6	120.3 (2)
O5—N6—C25	118.4 (2)	C27—C26—C25	118.4 (2)
O6—N6—C25	118.7 (2)	C27—C26—H26	120.8
O1—C1—C6	118.3 (2)	C25—C26—H26	120.8
O1—C1—C2	123.8 (2)	C26—C27—C22	122.1 (2)
C6—C1—C2	117.9 (2)	C26—C27—H27	119.0
C3—C2—C1	119.3 (2)	C22—C27—H27	119.0
C3—C2—C7	118.9 (2)	N2—C28—N5	109.2 (2)
C1—C2—C7	121.7 (2)	N2—C28—C23	127.6 (2)
C4—C3—C2	120.3 (2)	N5—C28—C23	123.1 (2)
C4—C3—H3	119.8	C30—C29—N2	108.06 (19)
C2—C3—H3	119.8	C30—C29—C31	128.5 (2)
C5—C4—C3	121.3 (2)	N2—C29—C31	123.5 (2)
C5—C4—N4	119.7 (2)	N5—C30—C29	106.2 (2)
C3—C4—N4	119.0 (2)	N5—C30—C37	120.6 (2)
C6—C5—C4	118.7 (2)	C29—C30—C37	133.1 (2)
C6—C5—H5	120.7	C36—C31—C32	119.5 (2)
C4—C5—H5	120.7	C36—C31—C29	120.0 (2)
C5—C6—C1	122.3 (2)	C32—C31—C29	120.4 (2)
C5—C6—H6	118.8	C33—C32—C31	119.9 (2)
C1—C6—H6	118.8	C33—C32—H32	120.0
N1—C7—N3	109.5 (2)	C31—C32—H32	120.0
N1—C7—C2	127.0 (2)	C34—C33—C32	120.0 (3)
N3—C7—C2	123.5 (2)	C34—C33—H33	120.0
C9—C8—N1	107.98 (19)	C32—C33—H33	120.0
C9—C8—C10	129.8 (2)	C33—C34—C35	120.4 (2)
N1—C8—C10	122.2 (2)	C33—C34—H34	119.8
N3—C9—C8	106.2 (2)	C35—C34—H34	119.8
N3—C9—C16	120.3 (2)	C34—C35—C36	119.7 (3)
C8—C9—C16	133.4 (2)	C34—C35—H35	120.2
C11—C10—C15	118.8 (2)	C36—C35—H35	120.2
C11—C10—C8	120.2 (2)	C35—C36—C31	120.4 (2)
C15—C10—C8	120.9 (2)	C35—C36—H36	119.8
C12—C11—C10	120.5 (2)	C31—C36—H36	119.8
C12—C11—H11	119.7	C42—C37—C38	119.3 (2)
C10—C11—H11	119.7	C42—C37—C30	119.6 (2)
C11—C12—C13	120.4 (3)	C38—C37—C30	121.1 (2)
C11—C12—H12	119.8	C39—C38—C37	120.0 (2)
C13—C12—H12	119.8	C39—C38—H38	120.0
C14—C13—C12	119.1 (2)	C37—C38—H38	120.0
C14—C13—H13	120.4	C38—C39—C40	120.5 (2)
C12—C13—H13	120.4	C38—C39—H39	119.8
C15—C14—C13	120.9 (3)	C40—C39—H39	119.8
C15—C14—H14	119.6	C41—C40—C39	119.6 (2)
C13—C14—H14	119.6	C41—C40—H40	120.2
C14—C15—C10	120.3 (2)	C39—C40—H40	120.2
C14—C15—H15	119.9	C40—C41—C42	120.2 (2)
C10—C15—H15	119.9	C40—C41—H41	119.9
C21—C16—C17	118.9 (2)	C42—C41—H41	119.9
C21—C16—C9	120.0 (2)	C37—C42—C41	120.4 (2)
C17—C16—C9	120.9 (2)	C37—C42—H42	119.8
C18—C17—C16	119.9 (2)	C41—C42—H42	119.8
C18—C17—H17	120.1	H1W—O1W—H2W	108.7 (16)
C16—C17—H17	120.1	H3W—O2W—H4W	111.1 (16)
			
Cu—O1—C1—C6	161.82 (17)	Cu—O2—C22—C27	164.29 (16)
Cu—O1—C1—C2	−18.7 (3)	Cu—O2—C22—C23	−15.6 (3)
O1—C1—C2—C3	178.5 (2)	O2—C22—C23—C24	176.6 (2)
C6—C1—C2—C3	−2.1 (3)	C27—C22—C23—C24	−3.3 (3)
O1—C1—C2—C7	−2.0 (4)	O2—C22—C23—C28	−2.7 (3)
C6—C1—C2—C7	177.4 (2)	C27—C22—C23—C28	177.4 (2)
C1—C2—C3—C4	0.4 (4)	C22—C23—C24—C25	3.2 (3)
C7—C2—C3—C4	−179.2 (2)	C28—C23—C24—C25	−177.5 (2)
C2—C3—C4—C5	1.2 (4)	C23—C24—C25—C26	−1.4 (3)
C2—C3—C4—N4	−179.3 (2)	C23—C24—C25—N6	179.3 (2)
O4—N4—C4—C5	164.7 (3)	O5—N6—C25—C24	−174.5 (2)
O3—N4—C4—C5	−13.4 (4)	O6—N6—C25—C24	4.7 (3)
O4—N4—C4—C3	−14.8 (4)	O5—N6—C25—C26	6.2 (3)
O3—N4—C4—C3	167.0 (2)	O6—N6—C25—C26	−174.6 (2)
C3—C4—C5—C6	−0.9 (4)	C24—C25—C26—C27	−0.3 (3)
N4—C4—C5—C6	179.6 (2)	N6—C25—C26—C27	179.0 (2)
C4—C5—C6—C1	−0.9 (4)	C25—C26—C27—C22	0.1 (3)
O1—C1—C6—C5	−178.1 (2)	O2—C22—C27—C26	−178.2 (2)
C2—C1—C6—C5	2.4 (4)	C23—C22—C27—C26	1.7 (3)
C8—N1—C7—N3	0.0 (3)	C29—N2—C28—N5	−0.2 (2)
Cu—N1—C7—N3	−175.88 (15)	Cu—N2—C28—N5	−174.71 (14)
C8—N1—C7—C2	178.9 (2)	C29—N2—C28—C23	176.4 (2)
Cu—N1—C7—C2	3.1 (3)	Cu—N2—C28—C23	1.9 (3)
C9—N3—C7—N1	−0.2 (3)	C30—N5—C28—N2	0.1 (3)
C9—N3—C7—C2	−179.2 (2)	C30—N5—C28—C23	−176.7 (2)
C3—C2—C7—N1	−170.4 (2)	C24—C23—C28—N2	−169.5 (2)
C1—C2—C7—N1	10.1 (4)	C22—C23—C28—N2	9.8 (4)
C3—C2—C7—N3	8.4 (4)	C24—C23—C28—N5	6.7 (3)
C1—C2—C7—N3	−171.1 (2)	C22—C23—C28—N5	−174.0 (2)
C7—N1—C8—C9	0.3 (3)	C28—N2—C29—C30	0.2 (2)
Cu—N1—C8—C9	175.74 (17)	Cu—N2—C29—C30	174.12 (16)
C7—N1—C8—C10	−178.3 (2)	C28—N2—C29—C31	179.8 (2)
Cu—N1—C8—C10	−2.8 (3)	Cu—N2—C29—C31	−6.3 (3)
C7—N3—C9—C8	0.4 (3)	C28—N5—C30—C29	0.0 (2)
C7—N3—C9—C16	−175.8 (2)	C28—N5—C30—C37	178.4 (2)
N1—C8—C9—N3	−0.4 (3)	N2—C29—C30—N5	−0.1 (2)
C10—C8—C9—N3	178.0 (2)	C31—C29—C30—N5	−179.7 (2)
N1—C8—C9—C16	175.1 (2)	N2—C29—C30—C37	−178.2 (2)
C10—C8—C9—C16	−6.6 (5)	C31—C29—C30—C37	2.2 (4)
C9—C8—C10—C11	141.4 (3)	C30—C29—C31—C36	140.7 (3)
N1—C8—C10—C11	−40.4 (3)	N2—C29—C31—C36	−38.8 (3)
C9—C8—C10—C15	−41.4 (4)	C30—C29—C31—C32	−37.7 (4)
N1—C8—C10—C15	136.8 (2)	N2—C29—C31—C32	142.9 (2)
C15—C10—C11—C12	−2.1 (4)	C36—C31—C32—C33	−0.6 (4)
C8—C10—C11—C12	175.1 (2)	C29—C31—C32—C33	177.8 (2)
C10—C11—C12—C13	0.8 (4)	C31—C32—C33—C34	0.0 (4)
C11—C12—C13—C14	1.0 (4)	C32—C33—C34—C35	0.7 (4)
C12—C13—C14—C15	−1.5 (4)	C33—C34—C35—C36	−0.7 (4)
C13—C14—C15—C10	0.2 (4)	C34—C35—C36—C31	0.0 (4)
C11—C10—C15—C14	1.6 (3)	C32—C31—C36—C35	0.6 (4)
C8—C10—C15—C14	−175.6 (2)	C29—C31—C36—C35	−177.8 (2)
N3—C9—C16—C21	−36.9 (3)	N5—C30—C37—C42	−47.8 (3)
C8—C9—C16—C21	148.2 (3)	C29—C30—C37—C42	130.1 (3)
N3—C9—C16—C17	139.6 (2)	N5—C30—C37—C38	132.7 (2)
C8—C9—C16—C17	−35.3 (4)	C29—C30—C37—C38	−49.4 (4)
C21—C16—C17—C18	2.1 (4)	C42—C37—C38—C39	−0.1 (4)
C9—C16—C17—C18	−174.5 (2)	C30—C37—C38—C39	179.4 (2)
C16—C17—C18—C19	0.4 (4)	C37—C38—C39—C40	−0.2 (4)
C17—C18—C19—C20	−2.3 (4)	C38—C39—C40—C41	0.2 (4)
C18—C19—C20—C21	1.7 (4)	C39—C40—C41—C42	0.2 (4)
C19—C20—C21—C16	0.8 (4)	C38—C37—C42—C41	0.5 (4)
C17—C16—C21—C20	−2.7 (4)	C30—C37—C42—C41	−179.0 (2)
C9—C16—C21—C20	173.9 (2)	C40—C41—C42—C37	−0.6 (4)

### Hydrogen-bond geometry (Å, º)

Hydrogen-bond geometry (Å, °) for (I). *Cg*1 is the ring centroid of the C16–C21 ring.

**Table:**

*D*—H···*A*	*D*—H	H···*A*	*D*···*A*	*D*—H···*A*
N3—H3*N*···O1*W*^i^	0.89 (2)	1.91 (2)	2.790 (3)	173 (3)
N5—H5*N*···O2*W*	0.88 (2)	1.95 (2)	2.822 (3)	172 (3)
O1*W*—H1*W*···O2	0.85 (2)	1.92 (2)	2.745 (2)	164 (2)
O1*W*—H2*W*···O2*W*^ii^	0.85 (2)	2.21 (2)	2.868 (2)	134 (2)
O2*W*—H3*W*···O1^ii^	0.84 (2)	2.01 (2)	2.841 (2)	172 (2)
O2*W*—H4*W*···O3^iii^	0.84 (2)	2.27 (2)	2.938 (2)	136 (2)
C3—H3···O1*W*^i^	0.95	2.57	3.435 (3)	151
C33—H33···O5^iv^	0.95	2.48	3.345 (3)	151
C5—H5···O6^v^	0.95	2.50	3.361 (3)	151
C34—H34···*Cg*1^vi^	0.95	2.49	3.426 (3)	168

Symmetry codes: (i) −*x*+2, −*y*+1, −*z*+1; (ii) −*x*+1, −*y*+1, −*z*+1; (iii) *x*−1, *y*, *z*−1; (iv) −*x*+1, *y*+1/2, −*z*+1/2; (v) *x*+1, *y*, *z*+1; (vi) *x*, −*y*+1/2, *z*−1/2.
